# PARP Inhibitors in Biliary Tract Cancer: A New Kid on the Block?

**DOI:** 10.3390/medicines7090054

**Published:** 2020-08-31

**Authors:** Angela Dalia Ricci, Alessandro Rizzo, Chiara Bonucci, Nastassja Tober, Andrea Palloni, Veronica Mollica, Ilaria Maggio, Marzia Deserti, Simona Tavolari, Giovanni Brandi

**Affiliations:** 1 Department of Experimental, Diagnostic and Specialty Medicine, S.Orsola-Malpighi Hospital, University of Bologna, 40128 Bologna, Italy; dalia.ricci@gmail.com (A.D.R.); chiarabonucci1@gmail.com (C.B.); nastassja@cheapnet.it (N.T.); andrepalloni@gmail.com (A.P.); veronica.mollica7@gmail.com (V.M.); ila.mag88@gmail.com (I.M.); giovanni.brandi@unibo.it (G.B.); 2 Center of Applied Biomedical Research, S. Orsola-Malpighi University Hospital, 40128 Bologna, Italy; marzia.deserti@gmail.com (M.D.); simona.tavolari@unibo.it (S.T.)

**Keywords:** biliary tract cancer, cholangiocarcinoma, PARP, BRCA, olaparib, rucaparib, liver cancer

## Abstract

Poly adenosine diphosphate-ribose polymerase inhibitors (PARPi) represent an effective therapeutic strategy for cancer patients harboring germline and somatic aberrations in DNA damage repair (DDR) genes. *BRCA1/2* mutations occur at 1–7% across biliary tract cancers (BTCs), but a broader spectrum of DDR gene alterations is reported in 28.9–63.5% of newly diagnosed BTC patients. The open question is whether alterations in genes that are well established to have a role in DDR could be considered as emerging predictive biomarkers of response to platinum compounds and PARPi. Currently, data regarding PARPi in BTC patients harboring BRCA and DDR mutations are sparse and anecdotal; nevertheless, a variety of clinical trials are testing PARPi as monotherapy or in combination with other anticancer agents. In this review, we provide a comprehensive overview regarding the genetic landscape of DDR pathway deficiency, state of the art and future therapeutic implications of PARPi in BTC, looking at combination strategies with immune-checkpoint inhibitors and other anticancer agents in order to improve survival and quality of life in BTC patients.

## 1. Introduction

**Figure 1:**
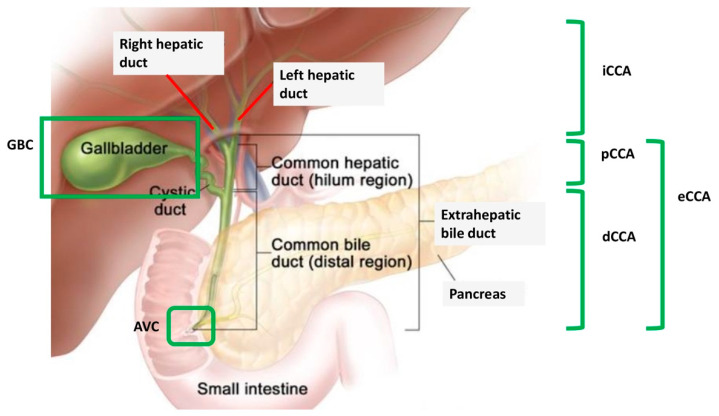
Anatomical subvariants of biliary tract cancer. AVC: ampulla of Vater cancer; dCCA: distal cholangiocarcinoma; eCCA: extrahepatic cholangiocarcinoma; GBC: gallbladder cancer; iCCA: intrahepatic cholangiocarcinoma; pCCA: perihilar cholangiocarcinoma.

Biliary tract cancers (BTCs) are a relatively rare group of malignancies arising from different anatomical locations of the biliary tree and including intrahepatic cholangiocarcinoma (iCCA), extrahepatic cholangiocarcinoma (eCCA), gallbladder cancer (GBC), and ampulla of Vater cancer (AVC) (Figure 1) [1,2]. 

BTC represents the second most frequent primary liver cancer after hepatocellular carcinoma (HCC), accounting for about 3% of all gastrointestinal tumors [3,4]. The incidence of BTC has increased in both western and eastern countries in the past two decades, concurrently with the rising incidence of iCCA, probably related to changes in tumor classification and better disease recognition [5]. Despite recent advances in the management of localized and metastatic disease, the prognosis of BTC patients remains dismal since the majority of cases are often diagnosed when unresectable or metastatic and the 5-year survival for patients with distant disease is about 5% [6]. To date, radical surgery is the only curative treatment option for BTC, but unfortunately, these malignancies are frequently asymptomatic in early stages, and approximately 40% of the patients considered resectable at the moment of diagnosis are found to be unresectable during exploratory laparotomy [7,8]. Systemic chemotherapy is the backbone of palliative treatment for BTC patients, with the combination of cisplatin plus gemcitabine representing the current standard of care in the front-line setting, following the results of the ABC-02 trial [9]. Although this phase III trial showed a survival advantage for cisplatin–gemcitabine over gemcitabine monotherapy, nearly all BTC patients develop progressive disease during first-line treatment, with a median overall survival (OS) of less than a year [10]. Thus, improving outcomes in patients affected by advanced/metastatic BTC represents an urgent need. 

In recent years, an unprecedented amount of genomic studies has begun to unveil the complex molecular landscape of BTC, shedding new light on novel therapeutic opportunities of this poor-prognosis malignancy and opening the era of tailor-made oncology in BTC [11]. In fact, the emergence of novel therapies is modifying previous treatment algorithms for BTC—especially in iCCA, where targeting isocitrate dehydrogenase (IDH) mutations and fibroblast growth factor receptor (FGFR) fusions is entering in clinical practice [12]. Comprehensive sequencing studies of BTC showed that nearly 40% of patients harbor a potentially targetable genetic alteration, emphasizing the genomic complexity of the disease, with several reports that have been focused on cell-cycle dysregulation, DNA damage repair (DDR) pathway deficiency, and genomic instability [13]. 

*BRCA1/2* are the most well-studied DDR genes, and their prevalence fluctuates from 1% to 7% in patients affected by BTC [13], with *BRCA2* suggested to be more frequent in GBC [14]. Although these mutations generally correlate with poor response to standard treatments, previous reports about BTC suggested a role for platinum salts and poly (ADP-ribose) polymerase-inhibitors (PARPi) as successful therapeutic options in somatic and/or germline *BRCA* mutations (*BRCAm*) carriers [15]. Evidence from phase III clinical trials has led to PARPi approval in breast and ovarian cancers, and the use of PARPi is going to be extended also to prostate and pancreatic cancer [16,17,18]. In fact, from the first launch of the PARPi olaparib in 2014, recent years have seen the FDA approval of other PARPi, including niraparib, rucaparib, and talazoparib in distinct settings [16,19]. More specifically, niraparib can be actually used as maintenance therapy in recurrent platinum-sensitive epithelial ovarian cancer following the results of the PRIMA/ENGOT-OV26/GOG-3012 trial [16]. In this randomized phase III trial, median progression-free survival (PFS) was significantly longer in the niraparib arm compared to that in the placebo group (21.9 versus 10.4 months) in patients affected by advanced ovarian cancer experiencing response to platinum-based chemotherapy. Similarly, many other PARPi have also entered clinical practice, as in the case of breast cancer where the OlympiAD and the EMBRACA trials have opened the doors of a new world, inaugurating the “PARPi Era” in HER2-negative *BRCAm* metastatic breast cancer [20,21]. According to OlympiAD—comparing olaparib monotherapy with single-agent chemotherapy of the physician’s choice (capecitabine, eribulin, or vinorelbine)—olaparib treatment provided a significant benefit in terms of PFS, with risk of disease progression or death 42% lower with olaparib single-agent than with chemotherapy [20]. With a study design similar to OlympiAD, the randomized phase III EMBRACA trial compared talazoparib versus standard single-agent chemotherapy of the physician’s choice (capecitabine, eribulin, gemcitabine, or vinorelbine) in advanced breast cancer patients with germline *BRCAm*, observing that talazoparib provided a statistically significant benefit in terms of PFS (8.6 versus 5.6 months; Hazard Ratio 0.54; 95% CI, 0.41–0.71, *p* < 0.001) [21]. Moreover, PARPi have shown an overall manageable safety profile, with hematological toxicity—mainly anemia—representing the most frequent adverse event [20,21]. In fact, incidence of grade 3–4 anemia has been reported to be around 19% in subjects receiving olaparib or rucaparib, 25% in niraparib, and 23% in patients treated with talazoparib [22] while neutropenia and thrombocytopenia ranges from 10% to 27%; thus, a strict monitoring on blood cell counts should be conducted in patients receiving these treatments [22]. 

As previously stated, previous experiences in ovarian and breast cancer have paved the way toward a number of trials testing PARPi in several tumors, with PARPi that are currently under active evaluation also in BRCA-mutated biliary malignancies [8,9,10]. However, a larger spectrum of genes that compromise DDR pathway has been reported to occur in up to 28.9% of patients with newly diagnosed BTC, and to date, the optimal therapeutic strategy in BTC tumors harboring Homologous Recombination Deficiency (HRD) alterations is yet to be defined [23]. 

In this review, we provide a comprehensive overview regarding the genetic landscape of DDR pathway deficiency, the emerging therapeutic role of PARPi in BTC, and current perspectives and possible future therapeutic implications of DDR alterations across BTC. 

## 2. HRD, the Role of PARP in DDR and Synthetic Lethality

**Figure 2:**
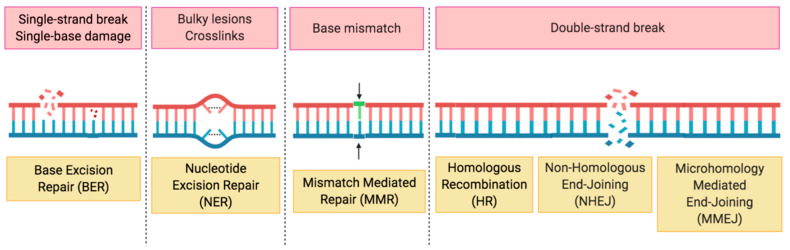
Overview of DNA repair mechanisms. BER: base excision repair; HR: homologous recombination; MMEJ: microhomology mediated end-joining; MMR: mismatch mediated repair; NER: nucleotide excision repair; NHEJ: non-homologous end-joining.

DNA damage and DNA repair, or lack thereof, have central importance in the induction of mutations. Additionally, since mutations drive the onset of nearly all malignancies, in physiological conditions, cells activate to defend themselves through a series of molecular pathways, the DDR, in order to handle genotoxic damage usually arising as single-strand breaks (SSBs) or double-strand breaks (DSBs) (Figure 2) [24]. 

Critical pathways able to fix DSBs are homologous recombination repair (HRR)—a form of DNA repair using homologous DNA sequences—microhomology mediated end-joining (MMEJ), and non-homologous end-joining (NHEJ), which conversely often leads to genetic material loss, thus resulting in genetic alterations [25,26]. Conversely, SSBs are mainly repaired by mechanisms such as base excision repair (BER), nucleotide excision repair (NER), or mismatch mediated repair (MMR) (Figure 2) [27,28]. Key elements in the DDR are the PARP enzymes, having an important role in SSBs repair and also taking part in HRR and NHEJ [29]. 

PARP (poly (ADP-ribose) polymerase) is a family of enzymes, including PARP1, PARP2, and PARP3 [30]. Interestingly, PARP1 is responsible for almost 80–90% of DDR activity, and in terms of structure, PARP1 presents a DNA binding domain at the N-terminus, with three zinc-finger-related domains able to recognize sites of damaged sequences [31]; moreover, PARP1 has a catalytic domain encompassing two subdomains: a helical domain and an ADP-ribosyltransferase catalytic transferring the ADP-ribose from NAD+ to protein residues, generating poly(ADP-ribose) chains (PAR) [32,33]. In fact, PARP1 and PARP2 are DNA damage sensors and signal transducers, able to synthesize branched PAR chains on target proteins through a process termed PARylation [34]. When PARP1 binds DNA, the catalytic function of PARP1 is activated following several allosteric modifications, leading to PARylation and recruitment of DNA repair effectors, including XRCC1 [35]. 

*BRCA1* and *BRCA2* are fundamental genes involved in HRR [36] and since they are critical in the process of DSBs repair, *BRCA1/2* germline mutations are associated with higher risk of carcinogenesis due to a mutational event on the other allele [37]. The same occurs when other genes essential for HRR are mutated, resulting in HRD [38,39,40]. 

**Figure 3:**
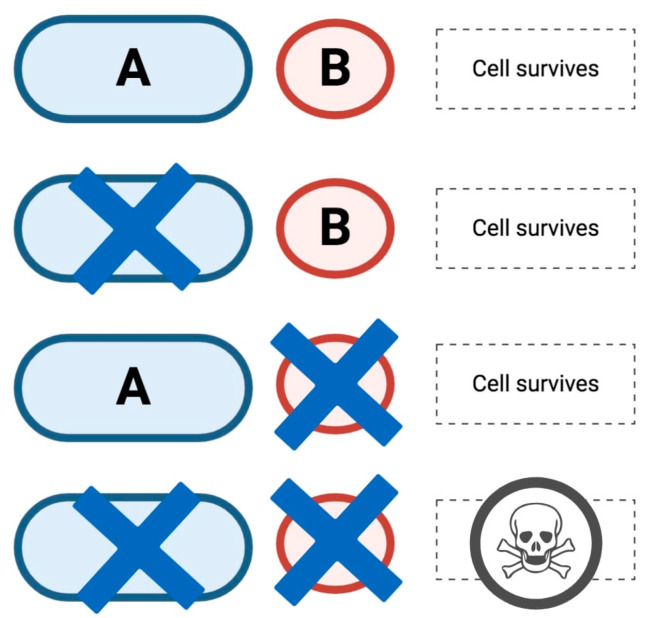
Schematic figure representing synthetic lethality. As outlined, the simultaneous alteration of gene *A* and gene *B* results in cell death while the alteration of either gene does not. When the concept of synthetic lethality is applied to poly adenosine diphosphate-ribose polymerase inhibitors (PARPi) treatment, gene *B* represents a candidate therapeutic target used to target cells with gene *A* dysfunctions.

PARPi are oral small-molecule inhibitors of PARP1, PARP2, and PARP3, whose action is based on synthetic lethality, a well-known concept proposed nearly a century ago [41,42]. As schematically represented in Figure 3, according to synthetic lethality the concurrent alteration of two different genes results in cell death while the alteration of a single gene does not. In the specific case of cancer treatment, with gene *A* representing a tumor suppressor gene or an oncogene, gene *B* could represent a candidate therapeutic target which may be used in order to target cells with *A* dysfunction.

The inhibition of PARP causes the persistence of SSBs, resulting in DSBs [43,44]. More specifically, there are two main mechanisms of action of PARPi, both responsible for their antiblastic effect. First, PARPi inhibit catalytic activity of the enzyme by avoiding both PARylation of the repair site and autoPARylation [45]. The second and even more significant mechanism is represented by PARP trapping activity; in fact, PARPi trap PARP at its DNA binding site preventing repair processes, hesitating in cell death by mitotic catastrophe [46,47]. Moreover, the inhibition of this pathway can force cells to use alternative damage repair systems, namely non-homologous recombination processes [48,49], which are more error-prone and can result in large-scale genomic rearrangements, and finally, in apoptotic cell death [50]. 

## 3. DDR Deficiency and *BRCAm* in BTC

The role of DDR alterations is still widely unknown in BTC and only few data about their clinical impact are currently available [51]. However, germline or somatic *BRCAm* are being increasingly reported due to the possibility to identify a distinct subgroup of carriers that may benefit from a personalized treatment strategy [52,53]. Curiously, *BRCAm* in BTC have been observed more frequently as somatic rather than as germline mutations [54]. 

The prevalence of DDR defects in BTC has been described in a range between 28.9% and 63.5%, and unfortunately, this range of frequencies depends on current lack of consensus regarding methods for testing and defining DDR alterations in BTC [54,55]. The recent evolution of sequencing technologies and the use of comprehensive gene sequencing panels has resulted in improved ability to detect variations in DDR genes, beyond *BRCA1*/*2* [56]. Nevertheless, two major limitations of these methods are represented by the unclear functional role of variants of unknown significance in DDR genes and the inability to identify epigenetic silencing of the same genes [57]. Moreover, the main open question is whether defects in genes that are well established to have a role in DDR could be considered as predictive biomarkers of response to platinum compounds and PARPi [58]. 

Another issue concerns how many germline and somatic pathogenic variants should be tested in order to identify “BRCAness” phenotypes [59]. A panel of 17 germline and somatic DDR gene alterations (*ATM, BAP1, BARD1, BLM, BRCA1, BRCA2, BRIP1, CHEK2, FAM175A, FANCA, FANCC, NBN, PALB2, RAD50, RAD51, RAD51C,* and *RTEL1*) in addition to *BRCAm* has been recently proposed in order to evaluate a correlation with genomic instability in patients affected by pancreatic ductal adenocarcinoma (PDAC), thereby excluding potential emerging DDR genes such as *ARID1A, ATR, ATRX, CHEK1, RAD51L1,* and *RAD51L3* [60]. Notably, mutations in *ARID1A* have been reported in up to 14% of cholangiocarcinomas (CCAs) [61], and interestingly, *ARID1A*—a chromatin remodeler of the *SWI/SNF* (Switch/Sucrose Non-Fermentable) family—probably contributes to recruiting and stabilizing the SWI/SNF complex at DSBs, thus regulating the DNA damage checkpoint [62,63]. Moreover, evidence from in vivo and in vitro studies suggested that *ARID1A* deficiency may sensitize cancer cells to PARPi [57]. Another gene involved in HR mechanisms is *BAP1*, a tumor suppressor gene and a deubiquitinase promoting DNA DSBs repair [64]. Yu and colleagues suggested that BAP1-deficient cells were sensitive to ionizing radiation and other agents that induce DNA DSBs [65], and additionally, *BAP1* mutant CCAs are likely to have poorer prognosis and a predisposition to bone metastasis development [66]. 

Patients with *BRCAm* are predisposed for BTC, as *BRCA1/2* alterations have been associated with early onset BTC [51,52,53,54]. More specifically, data from the early 2000s by the Breast Cancer Linkage Consortium (BCLC) suggested that *BRCA2-*carriers had higher relative risk (RR) of developing BTC than patients affected by infection with liver parasites, hepatitis C virus, and hepatitis B virus (RR 4.97, 95% confidence interval (CI) 1.50–16.52) [67]. 

Importantly, defective DNA repair enhances tumor heterogeneity and promotes tumor progression [68]. Hence, *BRCAm* generally correlate with poor response to standard treatments, although notable responses to platinum-based treatment or PARPi have been reported [69]. In 2017, Golan and colleagues published a retrospective analysis of 18 patients with confirmed *BRCAm* CCA [15]. Interestingly, the 44% of patients (8 of 18) had personal or family history of *BRCA*-associated malignancy (breast, ovarian, prostate, and pancreatic cancer) [15]. Overall, clinical germline testing for BTC risk is currently not recommended in clinical practice and more efforts are needed to better identify high-risk groups that might benefit from screening, further exploring, and eventually confirming the potential predictive and prognostic value of DDR gene alterations.

## 4. PARPi in BTC

Available data regarding PARPi in BTC patients harboring *BRCAm* and DDR mutations are sparse and anecdotal, with OS ranging from 11 to 65 months and sporadic cases of sustained response to PARPi, which have been reported [15,70,71,72]. As previously stated, although based on a small number of subjects, the multicenter retrospective study by Golan and colleagues suggested some clinical features of patients affected by BTC with germline and/or somatic *BRCAm* [15]. The study included 18 patients, 5 with germline *BRCA1/2m* and 13 with somatic mutations; interestingly, 13 patients were treated with platinum-based chemotherapy and 4 with PARPi. In terms of survival, BTC patients with stage I–stage II presented a median OS of 40.3 months (95% CI, 6.73–108.15) and of 25 months in stage III–stage IV BTC [15]. According to the results of this study, the presence of *BRCA1/2m* appeared to carry a more favorable prognosis since patients experienced a prolonged survival compared to historical data regarding BTC [15]. In a recent report by Chae et al., DDR gene mutations were observed in 55 out of 88 (63.5%) patients receiving first-line platinum-based chemotherapy for advanced BTC, with DDR gene mutations associated with longer OS (21.0 vs. 13.3 months, *p* = 0.009) and PFS (6.9 vs. 5.7 months, *p* = 0.013) after treatment with platinum salts [52]. This association between platinum sensitivity and DDR gene mutations has been widely described in other malignancies, including ovarian and breast cancer [73,74,75]. Platinum salts such as carboplatin and cisplatin exert their cytotoxic effects through distinct cellular mechanisms [76]; more specifically, after entrance into cells, platinum salts react with DNA generating monoadducts, inter- and intraDNA strand cross-links, and are able to cause SSBs and DSBs [77]. Consequently, DNA replication and transcription are blocked by this structural distortion, resulting in cell cycle arrest, cell apoptosis, and necrosis [78]. In physiological conditions, DNA lesions caused by platinum salts are properly repaired by DDR mechanisms; therefore, since platinum salts are DNA cross-linking agents, it is readily apparent that these compounds are more likely to be effective in *BRCAm* malignancies [79]. For example, higher rates of pathological complete response have been observed in *BRCAm*, triple negative breast cancer patients treated with neoadjuvant platinum salts compared to wild-type subjects [80]. Similarly, the randomized *TNT* trial highlighted a notable response rate and PFS benefit in metastatic *BRCAm* breast cancer patients receiving carboplatin compared to those receiving docetaxel [81]. This topic is particularly important if we look at BTC, where platinum-based chemotherapy represents the mainstay of palliative treatment following the results of the landmark ABC-02 trial and the more recent ABC-06 study [9,82,83]. 

To date, there is no evidence in literature regarding the efficacy of PARPi in BTC patients harboring DDR gene alterations, with the exception of a recent case report demonstrating a clinical benefit with olaparib monotherapy in a patient affected by gallbladder cancer with an Ataxia telangiectasia mutated (*ATM)*-inactivating mutation [84]. Following several trials assessing PARPi in breast cancer and ovarian cancer, recent studies have tested the role of PARPi in patients affected by HRD gastrointestinal malignancies, with the pivotal POLO trial, which has provided important data in this setting [71]. In fact, this randomized phase III trial has suggested a novel option for precision oncology in PDAC by evaluating the PARPi olaparib (300 mg twice daily) as maintenance in PDAC patients with *BRCAm* and whose disease had not progressed during first-line platinum-based chemotherapy [71]. Among the 154 enrolled patients, PFS was significantly longer in the olaparib maintenance arm compared to that in the placebo group, with 7.4 versus 3.8 months (Hazard Ratio 0.95; 95% CI 0.35–0.82, *p* = 0.004). Meanwhile, in analogy to previous reports in other solid malignancies, olaparib maintenance treatment has presented an acceptable and manageable safety profile, without a significant impact on quality of life [85]. More recently, a recent randomized phase II trial showed impressive response rates (75% and 64%, respectively) and survival in *BRCA1/2m* PDAC patients receiving platinum-base chemotherapy plus the PARPi veliparib or platinum-based chemotherapy alone as front-line treatment [86]. 

Considering the anatomical and histological analogies with PDAC, and in an attempt to translate this experience, multiple clinical trials are now evaluating the potential role of PARPi in metastatic BTC. We reviewed MEDLINE/PubMed and ClinicalTrial.gov for published or ongoing clinical trials evaluating the efficacy of PARPi in BTC until 20th July 2020. The medical subject heading terms used for PubMed search were ((olaparib[Title]) OR (veliparib[Title]) OR (rucaparib[Title]) OR (niraparib[Title]) OR (talazoparib[Title]) OR (PARP[Title])) AND ((biliary[Title]) OR (cholangiocarcinoma[Title]) OR (gallbladder[Title])). The medical subject headings terms used for the search in ClinicalTrials.gov were (“Recruiting or not yet recruiting” as status), (“biliary tract cancer”, “biliary tract neoplasm”, “cholangiocarcinoma”, “gallbladder cancer”, “Ampulla cancer” as condition/disease) and (“PARP”, “olaparib”, “veliparib”, “niraparib”, “rucaparib”, or “talazoparib” as other terms). Table 1 summarizes ongoing trials on PARPi in BTC registered on clinicaltrials.gov.

## 5. Future Directions

With the aim to provide novel effective combinations, several ongoing clinical trials are evaluating PARPi in combination with other agents, including cytotoxic chemotherapy, immune-checkpoint inhibitors (ICIs), and tyrosine kinase inhibitors (Table 1) [87]. 

Early preclinical reports have suggested that PARP1 is implicated in *STAT3* (Signal Transduced and Activator of Transcription 3) dephosphorylation, thus resulting in a reduced transcriptional activity of *STAT3* and lower PD-L1 expression [88]. Conversely, inhibiting PARP would clearly result in higher PD-L1 transcription in cancer cells and Programmed death-ligand 1(PD-L1) expression [88]. These preliminary findings have paved the way toward a number of studies assessing ICIs combined with PARPi in several malignancies since PARP inhibition has been suggested to increase tumor mutational burden, augmenting DNA damage processes and upregulating PD-L1 expression. The combination of PARPi with PD-1 inhibitors highlighted interesting response rates and a manageable safety profile in early reports evaluating this therapeutic strategy [89]. In a phase I trial assessing the PARPi pamiparib with the PD-1 inhibitor tislelizumab in 25 patients affected by advanced solid tumors, a response rate of 25% was observed, with two complete responses (4%) and eight partial responses (16%) [89]. Interestingly, this study included highly pretreated patients, with 14 out of 25 harboring a germline or somatic *BRCA1/2* mutation. More recently, the report by Spizzo and colleagues on 1292 tumor samples of BTC patients suggested a potential association between *BRCAm* and ICIs response, with tumor mismatch repair, microsatellite instability status, and PD-L1 overexpression associated with *BRCAm* [54]. 

Another interesting strategy is based on angiogenesis. In fact, hypoxia decelerates the downregulation of DNA repair processes, which in turn may result in genomic instability [90,91]. Therefore, the combination of PARPi and anti-angiogenic agents could enhance synthetic lethality, as witnessed in other solid malignancies such as ovarian cancer [92]. Unfortunately, acquired resistance to PARPi is a major issue in patients receiving these molecules, for which several potential mechanisms have been suggested, including the inactivation of the DNA repair proteins 53BP1 or REV7 [93,94]. Thus, novel drug combinations and treatment strategies able to overcome or at least delay the emergence of resistant clones are required [95]. PI3k/Akt, MAPK, and other mitogen signaling pathways have been related to reduction in *HR* repair, and consequently, have been associated with secondary resistance to PARPi [96]. As in the case of ICIs, preclinical and early clinical reports have suggested a possible synergistic activity provided by the combination of PI3k and MEK inhibitors plus PARPi [97,98], and further data are awaited. 

Lastly, another strategy could be based on targeting IDH, a therapeutic option that is entering into clinical practice, with *IDH1* and *IDH2* mutations occurring in about 20% of iCCA patients [99,100]. Interestingly, *IDH1* action relies on the conversion of isocitrate to alfa-ketoglutarate; in case of *IDH* mutations, alfa-ketoglutarate is transformed by *IDH1* into 2-hydroxyglutarate (2-HG), which plays a role in tumor progression [101,102]. Since preclinical reports have detected alterations in the *HR* pathway and an increased PARPi sensitivity in *IDH1*-mutated malignancies, the strategy of combining PARPi with IDH-targeted treatments is under evaluation in the subgroup of BTC patients harboring IDH mutations (Table 1) [103,104]. 

## 6. Conclusions

Unfortunately, patients with advanced/metastatic BTC have a dismal prognosis and few therapeutic options, and therefore, there is an urgent need for novel treatment strategies in this setting. If PARPi have shown meaningful activity in several solid tumors, further efforts are needed to define the role of these novel agents in BTC. A key point would certainly be the identification of which patients are most likely to benefit from PARPi monotherapy or combinations. In fact, combination strategies of PARPi with ICIs and other anticancer treatments are being tested and the results of these investigations are awaited, with the hope to increase the number of medical options and to improve survival and quality of life in BTC patients. 

## Figures and Tables

**Table 1:** Current ongoing trials involving PARP inhibitors in biliary tract cancer (BTC) registered on clinicaltrials.gov.

Clinical Trial	Design	Cohort	Agent(s)	DDR Defect Screenings	Primary Endpoint
NCT03212274	Phase II, single arm	Refractory, metastatic CCA with *IDH1* or *IDH2* mutation	Olaparib	no	ORR
NCT03207347 (UF-STO-ETI-001)	Phase II, non-randomized	CCA after prior standard systemic treatment	Niraparib	yes *	ORR
NCT03991832	Phase II, non-randomized	*IDH*-mutated BTC after no more than 2 previous treatments	Olaparib + durvalumab	no	ORR, DCR
NCT03878095	Phase II, single arm	CCA or other *IDH*-mutated solid tumors after prior standard treatment	Olaparib + ceralasertib	no	ORR
NCT03639935	Phase II, single arm	BTC after prior standard systemic treatment	Rucaparib + nivolumab	no	Proportion of patients alive and without radiological or clinical progression at 4 months
NCT04042831	Phase II, single arm	BTC with somatic/germline mutations in DDR genes after platinum-based chemotherapy	Olaparib	yes **	ORR
NCT03337087	Phase I–II, single arm	Metastatic BTC after no more than 1 line of prior therapy in the metastatic setting	Nal-IRI and 5-FU with rucaparib	yes, only for phase II (*HRD* or *BRCA1* or *BRCA2* or *PALB2*)	dose limiting toxicities, ORR
NCT04171700	Phase II, single arm	Unresectable, locally advanced, or metastatic solid tumor after first-line treatment (including ampullary cancer)	Rucaparib	yes ***	ORR

CCA: cholangiocarcinoma; DCR: disease control rate; DDR: DNA damage repair; 5-FU: F-fluorouracil; HRD: homologous recombination deficiency; IDH: isocitrate dehydrogenase; Nal-IRI: nanoliposomal irinotecan; ORR: overall response rate; * somatic/germline mutation of *ARID1A, ATM, ATR, BACH1 [BRIP1], BAP1, BARD1, BLM, CHEK1, CHEK2,CDK2, CDK4, ERCC, FAM175A, FEN1, IDH1, IDH2, MRE11A, NBN [NBS1], PALB2, POLD1, PRKDC [DNA-PK], PTEN, RAD50, RAD51, RAD52, RAD54, RPA1, SLX4, WRN*, or *XRCC*; ** somatic/germline mutation of *ATM, ATR, CHEK2, BRCA 1/2, RAD51, BRIP1, PALB2, PTEN, FANC, NBN, EMSY, MRE11, ARID1A*; *** deleterious mutation of *BRCA1, BRCA2, PALB2, RAD51C, RAD51D, BARD1, BRIP1, FANCA, NBN, RAD51, or RAD51B*.
